# Expression of The *αβ* T-Cell Receptor Is Necessary for The Generation of The Thymic Medulla

**DOI:** 10.1155/1993/56290

**Published:** 1993

**Authors:** Donald B. Palmer, Joanne L. Viney, Mary A. Ritter, Adrian C. Hayday, Michael J. Owen

**Affiliations:** 1 Imperial Cancer research fund P.O. Box 123 Lincoln's Inn Fields London WC2A 3PX UK; 2 Department of Immunology Royal Postgraduate Medical School Du Cane Rd London W12 ONN UK; 3 Department of Biology Yale University Kline Biology Tower 219 Prospect St. New Haven CT 06511 USA

**Keywords:** Thymus, medullary epithelial cells, *αβ*-cell receptor, TCR*α*-mutant mice

## Abstract

The architecture of the thymus of mice that congenitally fail to express the *αβ* T-cell receptor (TCR*αβ*) has been examined by immunohistology. In these mice, a defined mutation was introduced into the TCRc gene by homologous recombination. By using antibodies specific for cortical or medullary epithelium and for major histocompatibility complex antigens, the network of cortical epithelium in these mice was shown to be essentially unaltered in comparison with that of normal mice. In contrast, the thymic medulla was considerably reduced in size. This analysis shows that expression of the αβ TCR but not the *γδ* TCR is obligatory for establishing the thymic medulla and suggests that the growth of medullary epithelial cells may require contact with TCR*αβ*-expressing cells.


*Developmental Immunology*, 1993, Vol 3, pp. 175–179
Reprints available directly from the publisher
Photocopying permitted by license only

© 1993 Harwood Academic Publishers GmbH
Printed in the United States of America

### Expression of the $\alpha\beta$ T-Cell Receptor is Necessary for the Generation of the Thymic Medulla

DONALD B. PALMER,[†] JOANNE L. VINEY,[†] MARY A. RITTER,[‡] ADRIAN C. HAYDAY,[§] and MICHAEL J. OWEN[*†]

[†]*Imperial Cancer research fund, P.O. Box 123, Lincoln's Inn Fields, London WC2A 3PX, UK*
[‡]*Department of Immunology, Royal Postgraduate Medical School, Du Cane Rd, London W12 0NN, UK*
[§]*Department of Biology, Kline Biology Tower, Yale University, 219 Prospect St., New Haven CT 06511*

The architecture of the thymus of mice that congenitally fail to express the $\alpha\beta$ T-cell receptor (TCR$\alpha\beta$) has been examined by immunohistology. In these mice, a defined mutation was introduced into the TCR$\alpha$ gene by homologous recombination. By using antibodies specific for cortical or medullary epithelium and for major histocompatibility complex antigens, the network of cortical epithelium in these mice was shown to be essentially unaltered in comparison with that of normal mice. In contrast, the thymic medulla was considerably reduced in size. This analysis shows that expression of the $\alpha\beta$ TCR but not the $\gamma\delta$ TCR is obligatory for establishing the thymic medulla and suggests that the growth of medullary epithelial cells may require contact with TCR$\alpha\beta$-expressing cells.

KEYWORDS: Thymus, medullary epithelial cells, $\alpha\beta$ T-cell receptor, TCR$\alpha$-mutant mice.

### INTRODUCTION

The thymic microenvironment is essential for the differentiation of T cells. Prothymocytes migrate from sites of haematopoiesis into the thymus and there undergo a series of differentiation and expansion events, during which they express either $\alpha\beta$ or $\gamma\delta$ forms of the TCR (reviewed by von Boehmer, 1988). TCR$\alpha\beta$-expressing thymocytes undergo negative selection to remove autoreactive TCRs and positive selection to generate MHC-restricted TCRs. Both epithelial and dendritic cells are mediators of the selection processes, with the epithelial cells being particularly important in positive selection (Benoist and Mathis, 1989; Vukmanovic et al., 1992).

The thymic lobules of normal mice are arranged into an outer cortex and an inner medulla. In contrast to the normal thymus, the network of medullary epithelial cells is poorly developed in scid mice (Shores et al., 1991; Surh et al., 1992). However, injection of mature T cells or transfer of normal bone marrow cells restores this network of epithelial cells (Shores et al., 1991; Surh et al., 1992). These observations suggest that mature T cells may be necessary for the development of the thymic medulla.

We have previously generated mice that fail to express the $\alpha\beta$ TCR by disrupting the TCR$\alpha$ chain by homologous recombination (Philpott et al., 1992). In these mice, thymocytes arrest at the double-positive (CD4+CD8+) stage of development and fail to mature into single positive (CD4+ or CD8+) thymocytes. We also observed that the thymic medulla, characterized by its less dense cellularity in histological preparations, was undetectable morphologically. However, the basis for the disruption of the cellular architcture of the medulla was not elucidated. To determine whether this loss extended to the network of medullary epithelial cells or other cells in the microenvironment, thymi from these mice were analyzed using a series of monoclonal antibodies specific for thymic epithelial subsets, and major histocompatibility complex antigens. In the present study, we show that there is a drastic reduction of medulla epithelial cells, suggesting that contact with TCR$\alpha\beta$ cells are necessary for the maintenance of the thymic medulla.

### MATERIALS AND METHODS

### Mice

Mice used in this study were congenitally deficient in mature $\alpha\beta$ TCR (TCR$\alpha$−/−) expressing cells as

*Corresponding author.

described by Philpott et al. (1992). The control mice were wild type (WT) littermates expressing normal levels of the $\alpha\beta$ TCR.

### Immunohistochemistry

Thymi were removed from 4–8-week-old mice, snap frozen in liquid nitrogen, and stored at $-70\,^{\circ}C$. Five-micron frozen sections were cut from the tissue, air dried, fixed in acetone, and stained using the indirect immunoperoxidase method at room temperature. Slides were incubated with primary antibody for 1 hour and after washing in Tris-buffered saline (TBS), pH 7.6, were incubated for a further hour with biotinylated secondary antibody diluted in TBS containing 2% normal mouse serum. After washing for 20 min, the sections were incubated with avidinperoxidase (Sigma, UK) and visualized by adding diaminobenzidine (DAB, Sigma) (10 mg/15 ml with 12 $\mu$l of $H_2O_2$). Slides were counterstained with haematoxylin, dehydrated, and mounted using DPX mountant (BDH, UK). Positively staining cells were identified by the presence of a brown reaction product.

### Reagents

The rat monoclonal antibodies (mAb) used in this study were HB-51, anti-$K^b$,$D^b$ (Ozato and Sachs, 1981); M5/114, anti-$A\beta^b$ (Bhattacharya et al., 1981); ER-TR5, anti-medullary epithelial cells, the gift of Dr. J. Owen (van Vliet et al., 1984); NLDC-145, anti-cortical epithelial cells and dendritic cells, the gift of Dr. D. Gray (Kraal et al., 1986); 4F1, anti-cortical and IVC4, anti-medullary epithelial cells (Kanariou et al., 1989). The polyclonal rabbit anti-keratin antibody was from Dakopatts (Denmark). The second-layer antibodies used were biotinylated sheep anti-rat Ig (Amersham, UK) and biotinylated mouse anti-rabbit Ig (Sigma).

### RESULTS

### Thymic Epithelial Staining

Generation of mAbs raised against thymic epithelium has revealed molecular heterogeneity within the thymus microenvironment (Ritter and Crispe, 1992). The mAbs NLDC-145 and 4F1 recognize cortical epithelium, and NLDC-145 also weakly reacts against dendritic cells (Kraal et al., 1986; Kanariou et al., 1989). In contrast, the mAbs ER-TR5 and IVC4 react specifically with medullary epithelium (van Vliet et al., 1984; Kanariou et al., 1989). Although both sets of anti-cortical and anti-medullary epithelium antibodies were used, only staining with NLDC-145 and ER-TR5 are shown. Staining with either NLDC-145 or 4F1 on normal sections of thymus revealed a typical network of cortical epithelial cells, which are characterized by long thin processes separated by densely packed thymocytes (Fig. 1a). In contrast, the network of medullary epithelial cells in a normal thymus, as revealed by the mAbs ER-TR5 and IVC4, possessed shorter processes that were surrounded by fewer lymphoid cells (Fig. 1c).

The thymic cortex of TCR$\alpha$-/- mice showed no apparent differences in comparison with that of the normal cortex, using either NLDC-145 or 4F1 (Fig. 1b). In contrast, the mAbs ER-TR5 and IVC4 showed that the thymic medulla was considerably reduced in size in the TCR$\alpha$-/- mice (Fig. 1d). Staining with the anti-keratin antibody revealed the total network of epithelial cells in the thymus and confirmed these findings in both the wild type and TCR$\alpha$-/- mice (Figs. 1e and 1f). Macrophages were evenly distributed in the thymus of both control and TCR$\alpha$-/- mice (data not shown).

### MHC Antigen Expression

On the normal thymus MHC class II distribution in the cortex revealed a reticular network of epithelial cells, whereas the medulla showed confluent staining of MHC class II molecules (Fig. 2a). This pattern of staining was in agreement with that shown by Rouse et al. (1979). In the TCR$\alpha$ mutant mice, class II expression of the cortical epithelial cells was unaltered. However, only a small area of dense medullary class II staining was evident, underscoring the small size of the medulla in these mice (Fig. 2b). MHC class I staining produced a similar picture, although the overall expression is weaker, of dense staining in the medulla that was almost absent in the thymus from TCR$\alpha$-/- mice (data not shown).

### DISCUSSION

We have previously shown that the less dense cellularity characteristic of the thymic medulla was lacking in thymic sections from mice congenitally deficient in expression of the TCR$\alpha$ gene (Philpott et cells, veiled cells and interdigitating cells in the mouse recognised by a monoclonal antibody. J. Exp. Med. **163:** 981–997.

Mat I., Melcher D., and Ritter M.A. (1991). Epithelial cell expression of the interleukin 4 receptor complex. *In*: Lymphatic tissues and *in vivo* immune responses, Ezine S., Berrih-Aknin S., and Imhof B., Eds. (New York: Marcel Dekker), pp. 27–32.

Ozato K., and Sachs D.H. (1981). Monoclonal antibodies to mouse MHC antigens III. hybridoma antibodies reacting to antigens of the $H2^b$ haplotype reveal genetic control of isotype expression. J. Immunol. **126:** 317–321.

Philpott K.L., Viney J.L., Kay G., Rastan S., Gardiner E.M., Chae S., Hayday A.C., and Owen M.J. (1992). Lymphoid development in mice congenitally lacking T cell receptor $\alpha\beta$-expressing cells. Science **256:** 1448–1452.

Ritter M.A., and Crispe I.N. (1992). The thymus, Male D., Ed. Oxford, UK: IRL Press.

Rouse R.V., van Ewijk W., Jones P.P., and Weisman I.L. (1979). Expression of MHC antigens by mouse thymic dendritic cells. J. Immunol. **122:** 2508–2515.

Shores E.W., van Ewijk W., and Singer A. (1991). Disorganization and restoration of thymic medullary epithelial cells in T cell receptor-negative scid mice: Evidence that receptor-bearing lymphocytes influence maturation of the thymic microenvironment. Eur. J. Immunol. **21:** 1657–1661.

Surh C.D., Ernst B., and Sprent J. (1992). Growth of epithelial cells in the thymic medulla is under the control of mature T cells. J. Exp. Med. **176:** 611–616.

van Vliet E.M., Melis M., and van Ewijk W. (1984). Monoclonal antibodies to stromal cell types of the mouse. Eur. J. Immunol. **14:** 524–529.

von Boehmer H. (1988). The developmental biology of T lymphocytes. Ann. Rev. Immunol. **6:** 309–326.

Vukmanovic S., Grandea III A.G., Faas S.J., Knowles B.B., and Bevan M.J. (1992). Positive selection of T-lymphocytes induced by intrathymic injection of a thymic epithelial cell line. Nature **359:** 729–732.

